# Supplementary material for: “At the end of the day I’d still have to use a condom!”: A qualitative investigation of use of HIV pre-exposure prophylaxis among young gay and bisexual men in Nairobi, Kenya
Source: PLOS Glob Public Health. 2026 Jul 16;6(7):e0005282. doi: 10.1371/journal.pgph.0005282 (PMC13375007; doi:10.1371/journal.pgph.0005282)
Supplement: Attachment — Submitted filename: Response to Reviewers C.K 2.docx [file pgph.0005282.s003.docx]

**“At the end of the day I'd have to use a condom!”****: A qualitative investigation of the use of HIV pre-exposure prophylaxis among young gay and bisexual men in Nairobi, Kenya**

**Point-by-point response to reviewers:**

We sincerely thank the reviewers for scheduling time to review our manuscript, first when we submitted it and now as we submit the second revision. The feedback provided by the reviewers was kind, thoughtful and thorough. We hope that we have revised the manuscript to the reviewers’ satisfaction, and believe that the revisions have greatly improved the quality of our manuscript.

We acknowledge the confusion regarding the study’s data availability. However, data cannot be shared publicly because they are qualitative interviews which contain personal and potentially identifiable information from members of both a criminalized and stigmatized population, and participants have consented to publication of anonymized quotes only. Data are available from the corresponding authors on reasonable request for researchers who meet the criteria for access to confidential data, as determined by the approving ethics committees i.e. University of the Witwatersrand Human Research Ethics Committee Medical (via HREC-Medical.ResearchOffice@wits.ac.za) and University of Nairobi-Kenyatta National Hospital Ethics and Research Committee (via uonknh_erc@uonbi.ac.ke).

**Reviewer 1**

This is an excellent manuscript that makes a significant contribution to the literature on HIV prevention for young men who have sex with men in sub-Saharan Africa. The research is methodologically sound, the findings are important, and the manuscript is well-written. The authors should be commended for their diligent and comprehensive revisions, which have resulted in a high-quality paper that is ready for publication.

***Response:*** *Thank you for this very encouraging and thoughtful feedback. We sincerely appreciate your positive assessment of the manuscript and are glad that the revisions have strengthened the paper. Your comments are greatly valued, and we are pleased that the study makes a meaningful contribution to the literature on HIV prevention among young men who have sex with men in sub-Saharan Africa.*

**Reviewer 2**

The authors present a clear and interesting study of how PrEP is understood among gay, bisexual, and other men who have sex with men in Nairobi, Kenya. It is likely to be of interest to the journal readers. I am aware I am a 'new' reviewer on an already revised manuscript, so I have tried to focus my comments on the methodological components of this work.

***Response:*** *Thank you for your thoughtful and constructive feedback. In addition to addressing provided comments, we have corrected the identified typos and rephrased select terms to improve clarity and consistency throughout the text.*

1. **Comment:** The mention of interviews in Tanzania is a bit confusing; if I'm not mistaken, they are not included in this analysis, so suggest removing this reference.

***Response:*** *The reference on Tanzania’s study has been removed.*

2. **Comment:** The authors repeatedly mention 'purposive sampling' but have not specified the nature. If efforts were made to maximise sociodemographic diversity, identify certain experiences, or similar, then I would specify this information. I think purposive sampling is very appropriate here, but would just have appreciated a bit more detail on this approach.

***Response:*** *Thank you for pointing this out. The purposive sampling in this specific study was based on participants being HIV negative as explained in lines 144 - 147.*

3. **Comment:** Relatedly, the authors mention their sample size was selected as "sufficient to yield thematic saturation." I understand this concept was introduced following comments from a previous reviewer, but it is widely out-of-favour among qualitative methodologists. It is unlikely anything like 'saturation' could be achieved with 22 people, but either way justification of 'sample size' is a positivist tradition better suited to quantitative study. I suggest the authors remove this sentence entirely. If there is some kind of need to 'justify' the sample, I would focus on its diversity, novelty, exploratory nature, or some other feature more aligned with the paradigms of qualitative inquiry.

***Response:*** *Thank you for the thoughtful suggestion. The mention on thematic saturation has since been removed.*

4. **Comment:** I will not suggest a total reimagining of the analysis, but I must point out the results as presented do not appear to be of a thematic analysis. Themes must contain some kind of higher-level organisation of a corpus, one that goes beyond simply presenting what participants say. The latter is not bad, but it is not thematic; it is also what it appears the authors have done, organising the data as answers to the main questions asked of participants (as defined on p8). I would, therefore, strongly encourage the authors to rename their analysis as 'qualitative description' or a similar technique more aligned with their approach

***Response:*** *Data analysis has since been revised and currently align with qualitative description as explained on page 8 from line 178 – 180.*

5. **Comment:** I suggest greater engagement with the quotations as presented. Typically, the authors present them as evidence of a particular point, but offer no follow-up including to situate them within a larger context. For example, I note the last quote from 'Norman' (p18) indicates there are no MSM-specific clinics in Mombasa where one can access PrEP, but in fact there are several! While I understand this is the 'truth' as understood by this participant, this point is not properly contextualised. Adding 1-2 sentences after this and similar quotes will help situate these data within a larger context.

***Response:*** *Thank you for the suggestion.* *We have since added follow-up sentences to quotes for contextualization such as in lines 282 – 284, 302 – 303, 310 – 311, 336 – 337, 368 – 369, and 383 – 385.*

6. **Comment:** While I agree criminalisation (as a structural expression of stigma) is likely to have played a role here, this point is not really supported by the data as presented. Is there a quotation that can better support this linkage? If not, I would perhaps rephrase the opening sentence of line 435, using 'discrimination' as a foundation with which to link to larger systems that oppress gay, bisexual, and other men who have sex with men in Kenya

***Response:*** *We agree with the reviewer that there were no reported incidences of criminalization among participants. The paragraph’s focus has therefore been changed from discrimination and criminalization to solely discrimination.*

7. **Comment:** Relatedly, the discussion on injectable PrEP is appropriate, but how does the existence of ongoing stigma (and clear preference for population-specific services) align with the federal government's focus on 'integration' as the preferred model for HIV prevention and management? As this is an important topic in the current discourse of Kenya's HIV health systems, 1-2 sentences on this point may be valuable.

***Response:*** *Thank you for this insightful comment.* *This has been included from lines 509 – 513.*
