# Supplementary material for: “At the end of the day I’d still have to use a condom!”: A qualitative investigation of use of HIV pre-exposure prophylaxis among young gay and bisexual men in Nairobi, Kenya
Source: PLOS Glob Public Health. 2026 Jul 16;6(7):e0005282. doi: 10.1371/journal.pgph.0005282 (PMC13375007; doi:10.1371/journal.pgph.0005282)
Supplement: Attachment — Submitted filename: Response to Reviewers C.K.docx [file pgph.0005282.s002.docx]

**“At the end of the day I'd have to use a condom!”****: A qualitative investigation of the use of HIV pre-exposure prophylaxis among young gay and bisexual men in Nairobi, Kenya**

**Point-by-point response to reviewers:**

We sincerely thank the reviewers for scheduling time to review our manuscript, first when we submitted it and now as we submit the revisions. The feedback provided by the reviewers was kind, thoughtful and thorough. We hope that we have revised the manuscript to the reviewers’ satisfaction, and believe that the revisions have greatly improved the quality of our manuscript.

**Reviewer 1**

1. **Comment:** Elaboration on Data Analysis Process: The methods section states that the IMB model guided data analysis and that thematic analysis was used. This is appropriate, but the manuscript would be strengthened by providing more explicit detail on how the IMB model was operationalized during the analysis. For example:
   ◦ Was the coding process primarily deductive, with codes derived from the IMB model constructs (Information, Motivation, Behavioral Skills), or was it more inductive, with themes emerging from the data that were then mapped onto the model?
   ◦ Could you provide a brief description of the coding process? For instance, how many researchers coded the transcripts, and how were discrepancies resolved to ensure inter-coder reliability?
   Adding this detail would enhance the transparency and replicability of the analytical process, which is a key criterion for PLOS journals.

***Response:*** *Clarification on how the IMB model was operationalized has been added on the manuscript (lines 183-185).*

*Please see lines 180-183 on the coding process and the number of researchers responsible for coding the data and how they ensured validity and reliability of the data.*

2. **Comment:** Justification of Sample Size and Data Saturation: The study includes 22 participants, which is a reasonable sample size for a qualitative study. However, the methods section would be more robust if it included a statement on data saturation. Were interviews conducted until thematic saturation was reached? If so, a brief statement explaining that no new major themes were emerging in the final interviews would provide a strong justification for the sample size and increase confidence in the comprehensiveness of the findings.

***Response:*** *Thank you for pointing this out. The selection of 22 out of the 52 participants was based on pragmatic considerations from the researchers. Thematic saturation was justified through previous successful studies with a similar sample size. This has now been clearly outlined on the manuscript lines 149-152.*

3. **Comment:** Structure of the Discussion: The discussion section is well-written and effectively contextualizes the findings. To further enhance its impact, consider structuring the initial part of the discussion more explicitly around the IMB model. For example, you could have sub-sections for Information-related findings, Motivation-related findings, and Behavioral Skills-related findings, discussing how your results align with or expand upon existing literature within each of these domains. This would create a very clear and powerful link back to your theoretical framework.

***Response:*** *Thank you for the thoughtful suggestion. The discussion section (line 371 - 502) has been revised to include sub-titles on content related to the IMB constructs and the newer PrEP modalities.*

4. **Comment:** Clarity on PrEP Modalities: The introduction and abstract mention both daily and on-demand oral PrEP. It would be helpful if the results section could briefly clarify whether participants distinguished between these two modalities in their discussions of barriers and facilitators. Were the concerns about stigma, for example, equally applicable to both daily and on-demand use? This is a minor point, but it could add another layer of nuance to the findings.

***Response:*** *Participants’ experiences and perceptions were largely confined to the daily regimen, which limited the researchers’ ability to fully assess views on challenges such as stigma toward on-demand PrEP. However, the manuscript includes results on YMSM’s knowledge and reaction toward on-demand Prep in lines 231.*

5. **Comment:** Title: The title is engaging and effectively uses a participant quote. This is a stylistic point, but you may consider whether a slightly more concise main title could also work. The current title is perfectly acceptable, so this is merely a suggestion for the authors to consider.

***Response:*** *We agree with the reviewer and have since tried to make the title more concise by leaving out the words ‘knowledge,’ ‘motivations,’ and ‘barriers.’ The current title reads; ‘“At the end of the day I'd have to use a condom!”: A qualitative investigation of the use of HIV pre-exposure prophylaxis among young gay and bisexual men in Nairobi, Kenya’*

**Overall comment:**

This is a well-written and timely manuscript that addresses a significant issue in global public health: the underutilization of PrEP among young men who have sex with men (YMSM) in Kenya. The study is well-grounded in the Information-Motivation-Behavioural Skills (IMB) model, the qualitative methodology is appropriate for the research question, and the findings provide valuable insights into the complex interplay of knowledge, motivations, and barriers influencing PrEP use in this key population. The manuscript is a substantial contribution to the field and is well-suited for publication in PLOS Global Public Health.

In summary, this is an excellent manuscript that makes a valuable contribution to the literature on HIV prevention for YMSM in sub-Saharan Africa. The research is sound, the findings are essential, and the paper is well-written. Addressing the minor points above will further enhance its clarity, rigor, and impact. I congratulate the authors on this vital work.

***Response:*** *Thank you for this feedback.*
