# Supplementary material for: “At the end of the day I’d still have to use a condom!”: A qualitative investigation of use of HIV pre-exposure prophylaxis among young gay and bisexual men in Nairobi, Kenya
Source: PLOS Glob Public Health. 2026 Jul 16;6(7):e0005282. doi: 10.1371/journal.pgph.0005282 (PMC13375007; doi:10.1371/journal.pgph.0005282)
Supplement: Attachment — Submitted filename: Response_to_Reviewers_C.K_auresp_3.docx [file pgph.0005282.s004.docx]

**“At the end of the day I'd have to use a condom!”****: A qualitative investigation of the use of HIV pre-exposure prophylaxis among young gay and bisexual men in Nairobi, Kenya**

**Point-by-point response to reviewers:**

We sincerely thank the reviewers for scheduling time to review our manuscript, first when we submitted it and now as we submit the final revision. The feedback provided by the reviewers was kind, thoughtful and thorough. We confirm that we have revised the manuscript to the reviewers’ satisfaction, and believe that the revisions have greatly improved the quality of our manuscript.

**Reviewer 1**

I commend the authors for the thorough and thoughtful revisions made to the manuscript. You addressed each of my comments with clarity and depth, and the additions, particularly the expanded methodological detail, justification of sample size, and restructuring of the discussion around the IMB model, have strengthened the manuscript considerably. The revisions enhance transparency, theoretical alignment, and overall coherence. I appreciate the care and responsiveness demonstrated throughout this process.

***Response:*** *Thank you for this very encouraging and thoughtful feedback. We sincerely appreciate your positive assessment of the manuscript and are glad that the revisions have strengthened the paper. Your comments are greatly valued, and we are pleased that the study makes a meaningful contribution to the literature on HIV prevention among young men who have sex with men in sub-Saharan Africa.*

**Reviewer 2**

I thank the authors for attending so carefully to my feedback. One final point is that the manuscript still repeatedly mentions ‘themes’ and ‘sub-themes’ despite the analytic shift away from thematic analysis. To avoid confusing the reader or inadvertently suggesting this was a thematic analysis, I would remove all such references and replace them with ‘categories’, ‘topics’, or similar. The idea of a qualitative descriptive analysis of this kind is that the data are organised around explicit questions or topics; instead of saying “…following sections explore the main themes that were defined from analysis”, it is probably more true to say “…following sections are organised around the primary topics explored through our interviews”. Could draw upon the IBM framework to give this shape. Once this final issue of semantics is resolved, I have no further feedback.

***Response:*** *Thank you for your thoughtful and constructive feedback. We have revised the manuscript to remove references to “themes” and “sub-themes” and replaced them with terminology consistent with a qualitative descriptive approach, including “categories” and “topics.” We have also updated the wording throughout to better reflect an analysis organised around the IMB framework and to avoid any implication of thematic analysis.*
