# Supplementary material for: “At the end of the day I’d still have to use a condom!”: A qualitative investigation of use of HIV pre-exposure prophylaxis among young gay and bisexual men in Nairobi, Kenya
Source: PLOS Glob Public Health. 2026 Jul 16;6(7):e0005282. doi: 10.1371/journal.pgph.0005282 (PMC13375007; doi:10.1371/journal.pgph.0005282)
Supplement: Peer review history

# Peer review: Decision Letter 0

**DOI:** 10.1371/journal.pgph.0005282.r001

**Authors:** Helen Howard

4 Dec 2025

PGPH-D-25-02608

“At the end of the day I'd have to use a condom!”: A qualitative investigation of knowledge, motivations, and barriers to use of HIV pre-exposure prophylaxis among young gay and bisexual men in Nairobi, Kenya

PLOS Global Public Health

Dear Dr. Cheruiyot,

Thank you for submitting your manuscript to PLOS Global Public Health. After careful consideration, we feel that it has merit but does not fully meet PLOS Global Public Health’s publication criteria as it currently stands. Therefore, we invite you to submit a revised version of the manuscript that addresses the points raised during the review process.

Please note that we have only been able to secure a single reviewer to assess your manuscript. We are issuing a decision on your manuscript at this point to prevent further delays in the evaluation of your manuscript. Please be aware that the editor who handles your revised manuscript might find it necessary to invite additional reviewers to assess this work once the revised manuscript is submitted. However, we will aim to proceed on the basis of this single review if possible.

Could you please revise the manuscript to carefully address the concerns raised?

Please submit your revised manuscript by Jan 17 2026 11:59PM. If you will need more time than this to complete your revisions, please reply to this message or contact the journal office at globalpubhealth@plos.org. When you're ready to submit your revision, log on to https://www.editorialmanager.com/pgph/ and select the 'Submissions Needing Revision' folder to locate your manuscript file.

Please include the following items when submitting your revised manuscript:

- A rebuttal letter that responds to each point raised by the editor and reviewer(s). You should upload this letter as a separate file labeled 'Response to Reviewers'.
- A marked-up copy of your manuscript that highlights changes made to the original version. You should upload this as a separate file labeled 'Revised Manuscript with Track Changes'.
- An unmarked version of your revised paper without tracked changes. You should upload this as a separate file labeled 'Manuscript'.

Guidelines for resubmitting your figure files are available below the reviewer comments at the end of this letter.

We look forward to receiving your revised manuscript.

Kind regards,

Helen Howard

Staff Editor

PLOS Global Public Health

Journal Requirements:

1. Please amend your detailed online Financial Disclosure statement. This is published with the article. It must therefore be completed in full sentences and contain the exact wording you wish to be published.

a) State the initials, alongside each funding source, of each author to receive each grant. For example: “This work was supported by the National Institutes of Health (####### to AM; ###### to CJ) and the National Science Foundation (###### to AM).”

b) State what role the funders took in the study. If the funders had no role in your study, please state: “The funders had no role in study design, data collection and analysis, decision to publish, or preparation of the manuscript.”

For more information, please go to our submission guidelines:

https://journals.plos.org/globalpublichealth/s/submission-guidelines#loc-financial-disclosure-statement

2. Please ensure that the funders and grant numbers match between the Financial Disclosure field and the Funding Information tab in your submission form. Note that the funders must be provided in the same order in both places as well.

3. Please update your online Competing Interests statement. If you have no competing interests to declare, please state: “The authors have declared that no competing interests exist.”

4. If the reviewer comments include a recommendation to cite specific previously published works, please review and evaluate these publications to determine whether they are relevant and should be cited. There is no requirement to cite these works unless the editor has indicated otherwise.

Additional Editor Comments (if provided):

[Note: HTML markup is below. Please do not edit.]

Reviewers' comments:

Reviewer's Responses to Questions

**Comments to the Author**

1. Does this manuscript meet PLOS Global Public Health’s publication criteria? Is the manuscript technically sound, and do the data support the conclusions? The manuscript must describe methodologically and ethically rigorous research with conclusions that are appropriately drawn based on the data presented.

Reviewer #1: Yes

**********

2. Has the statistical analysis been performed appropriately and rigorously?

Reviewer #1: N/A

**********

3. Have the authors made all data underlying the findings in their manuscript fully available (please refer to the Data Availability Statement at the start of the manuscript PDF file)?

The PLOS Data policy requires authors to make all data underlying the findings described in their manuscript fully available without restriction, with rare exception. The data should be provided as part of the manuscript or its supporting information, or deposited to a public repository. For example, in addition to summary statistics, the data points behind means, medians and variance measures should be available. If there are restrictions on publicly sharing data—e.g. participant privacy or use of data from a third party—those must be specified.

Reviewer #1: Yes

**********

4. Is the manuscript presented in an intelligible fashion and written in standard English?

PLOS Global Public Health does not copyedit accepted manuscripts, so the language in submitted articles must be clear, correct, and unambiguous. Any typographical or grammatical errors should be corrected at revision, so please note any specific errors here.

Reviewer #1: Yes

**********

5. Review Comments to the Author

Please use the space provided to explain your answers to the questions above. You may also include additional comments for the author, including concerns about dual publication, research ethics, or publication ethics. (Please upload your review as an attachment if it exceeds 20,000 characters)

Reviewer #1: This is a well-written and timely manuscript that addresses a significant issue in global public health: the underutilization of PrEP among young men who have sex with men (YMSM) in Kenya. The study is well-grounded in the Information-Motivation-Behavioural Skills (IMB) model, the qualitative methodology is appropriate for the research question, and the findings provide valuable insights into the complex interplay of knowledge, motivations, and barriers influencing PrEP use in this key population. The manuscript is a substantial contribution to the field and is well-suited for publication in PLOS Global Public Health.

My recommendation is for Minor Revision. The study is methodologically sound, and the conclusions are well-supported by the data. The suggested revisions below are intended to further strengthen the manuscript by adding clarity and depth to the methods and discussion sections.

Primary Areas for Revision

(Note: These are framed as major points but are addressable through textual revisions and do not require new data collection.)

1 Elaboration on Data Analysis Process: The methods section states that the IMB model guided data analysis and that thematic analysis was used. This is appropriate, but the manuscript would be strengthened by providing more explicit detail on how the IMB model was operationalized during the analysis. For example:

◦ Was the coding process primarily deductive, with codes derived from the IMB model constructs (Information, Motivation, Behavioral Skills), or was it more inductive, with themes emerging from the data that were then mapped onto the model?

◦ Could you provide a brief description of the coding process? For instance, how many researchers coded the transcripts, and how were discrepancies resolved to ensure inter-coder reliability?

Adding this detail would enhance the transparency and replicability of the analytical process, which is a key criterion for PLOS journals.

2 Justification of Sample Size and Data Saturation: The study includes 22 participants, which is a reasonable sample size for a qualitative study. However, the methods section would be more robust if it included a statement on data saturation. Were interviews conducted until thematic saturation was reached? If so, a brief statement explaining that no new major themes were emerging in the final interviews would provide a strong justification for the sample size and increase confidence in the comprehensiveness of the findings.

Minor Points for Revision

3 Structure of the Discussion: The discussion section is well-written and effectively contextualizes the findings. To further enhance its impact, consider structuring the initial part of the discussion more explicitly around the IMB model. For example, you could have sub-sections for Information-related findings, Motivation-related findings, and Behavioral Skills-related findings, discussing how your results align with or expand upon existing literature within each of these domains. This would create a very clear and powerful link back to your theoretical framework.

4 Clarity on PrEP Modalities: The introduction and abstract mention both daily and on-demand oral PrEP. It would be helpful if the results section could briefly clarify whether participants distinguished between these two modalities in their discussions of barriers and facilitators. Were the concerns about stigma, for example, equally applicable to both daily and on-demand use? This is a minor point, but it could add another layer of nuance to the findings.

5 Title: The title is engaging and effectively uses a participant quote. This is a stylistic point, but you may consider whether a slightly more concise main title could also work. The current title is perfectly acceptable, so this is merely a suggestion for the authors to consider.

In summary, this is an excellent manuscript that makes a valuable contribution to the literature on HIV prevention for YMSM in sub-Saharan Africa. The research is sound, the findings are essential, and the paper is well-written. Addressing the minor points above will further enhance its clarity, rigor, and impact. I congratulate the authors on this vital work.

**********

6. PLOS authors have the option to publish the peer review history of their article (what does this mean?). If published, this will include your full peer review and any attached files.

**Do you want your identity to be public for this peer review?**  If you choose “no”, your identity will remain anonymous but your review may still be made public.

For information about this choice, including consent withdrawal, please see our Privacy Policy.

Reviewer #1: **Yes:**  SANNI, Olaniyi Felix

**********

[NOTE: If reviewer comments were submitted as an attachment file, they will be attached to this email and accessible via the submission site. Please log into your account, locate the manuscript record, and check for the action link "View Attachments". If this link does not appear, there are no attachment files.]

-->Figure Resubmissions:

While revising your submission, we strongly recommend that you use PLOS’s NAAS tool (https://ngplosjournals.pagemajik.ai/artanalysis) to test your figure files. NAAS can convert your figure files to the TIFF file type and meet basic requirements (such as print size, resolution), or provide you with a report on issues that do not meet our requirements and that NAAS cannot fix.

After uploading your figures to PLOS’s NAAS tool - https://ngplosjournals.pagemajik.ai/artanalysis, NAAS will process the files provided and display the results in the "Uploaded Files" section of the page as the processing is complete. If the uploaded figures meet our requirements (or NAAS is able to fix the files to meet our requirements), the figure will be marked as "fixed" above. If NAAS is unable to fix the files, a red "failed" label will appear above. When NAAS has confirmed that the figure files meet our requirements, please download the file via the download option, and include these NAAS processed figure files when submitting your revised manuscript.

# Author comment: Author response to Decision Letter 1

**DOI:** 10.1371/journal.pgph.0005282.r002

17 Jan 2026

Attachment Submitted filename: Response to Reviewers C.K.docx

# Peer review: Decision Letter 1

**DOI:** 10.1371/journal.pgph.0005282.r003

**Authors:** Helen Howard, Jen Edwards

5 Mar 2026

PGPH-D-25-02608R1

“At the end of the day I'd have to use a condom!”: A qualitative investigation of use of HIV pre-exposure prophylaxis among young gay and bisexual men in Nairobi, Kenya

PLOS Global Public Health

Dear Dr. Cheruiyot,

Thank you for submitting your manuscript to PLOS Global Public Health. After careful consideration, we feel that it has merit but does not fully meet PLOS Global Public Health’s publication criteria as it currently stands. Therefore, we invite you to submit a revised version of the manuscript that addresses the points raised during the review process.

The manuscript has been evaluated by two reviewers, and their comments are available below. Reviewer 2 raises a number of concerns regarding the detail provided on some of the approach and results. Could you please carefully revise the manuscript to address all comments raised?

We also note that Reviewer 2 disagrees with previous comments regarding justification of sample sizes. Please consider the comments from both reviewers in the context of your research and current methodological norms. We ask that you revise the manuscript as you feel most appropriate and provide a comment or rebuttal on your decision in your response to reviewers.

Please submit your revised manuscript by Apr 19 2026 11:59PM. If you will need more time than this to complete your revisions, please reply to this message or contact the journal office at globalpubhealth@plos.org. When you're ready to submit your revision, log on to https://www.editorialmanager.com/pgph/ and select the 'Submissions Needing Revision' folder to locate your manuscript file.

Please include the following items when submitting your revised manuscript:

- A letter that responds to each point raised by the editor and reviewer(s). You should upload this letter as a separate file labeled 'Response to Reviewers'.
- A marked-up copy of your manuscript that highlights changes made to the original version. You should upload this as a separate file labeled 'Revised Manuscript with Track Changes'.
- An unmarked version of your revised paper without tracked changes. You should upload this as a separate file labeled 'Manuscript'.

Guidelines for resubmitting your figure files are available below the reviewer comments at the end of this letter.

We look forward to receiving your revised manuscript.

Kind regards,

Jen Edwards

Staff Editor

PLOS Global Public Health

Journal Requirements:

If the reviewer comments include a recommendation to cite specific previously published works, please review and evaluate these publications to determine whether they are relevant and should be cited. There is no requirement to cite these works unless the editor has indicated otherwise.

Additional Editor Comments (if provided):

[Note: HTML markup is below. Please do not edit.]

Reviewers' comments:

Reviewer's Responses to Questions

**Comments to the Author**

1. If the authors have adequately addressed your comments raised in a previous round of review and you feel that this manuscript is now acceptable for publication, you may indicate that here to bypass the “Comments to the Author” section, enter your conflict of interest statement in the “Confidential to Editor” section, and submit your "Accept" recommendation.

Reviewer #1: All comments have been addressed

Reviewer #2: (No Response)

**********

2. Does this manuscript meet PLOS Global Public Health’s publication criteria? Is the manuscript technically sound, and do the data support the conclusions? The manuscript must describe methodologically and ethically rigorous research with conclusions that are appropriately drawn based on the data presented.

Reviewer #1: Yes

Reviewer #2: Yes

**********

3. Has the statistical analysis been performed appropriately and rigorously?

Reviewer #1: Yes

Reviewer #2: N/A

**********

4. Have the authors made all data underlying the findings in their manuscript fully available (please refer to the Data Availability Statement at the start of the manuscript PDF file)?

The PLOS Data policy requires authors to make all data underlying the findings described in their manuscript fully available without restriction, with rare exception. The data should be provided as part of the manuscript or its supporting information, or deposited to a public repository. For example, in addition to summary statistics, the data points behind means, medians and variance measures should be available. If there are restrictions on publicly sharing data—e.g. participant privacy or use of data from a third party—those must be specified.

Reviewer #1: Yes

Reviewer #2: No

**********

5. Is the manuscript presented in an intelligible fashion and written in standard English?

PLOS Global Public Health does not copyedit accepted manuscripts, so the language in submitted articles must be clear, correct, and unambiguous. Any typographical or grammatical errors should be corrected at revision, so please note any specific errors here.

Reviewer #1: Yes

Reviewer #2: Yes

**********

6. Review Comments to the Author

Please use the space provided to explain your answers to the questions above. You may also include additional comments for the author, including concerns about dual publication, research ethics, or publication ethics. (Please upload your review as an attachment if it exceeds 20,000 characters)

Reviewer #1: This is an excellent manuscript that makes a significant contribution to the literature on HIV prevention for young men who have sex with men in sub-Saharan Africa. The research is methodologically sound, the findings are important, and the manuscript is well-written. The authors should be commended for their diligent and comprehensive revisions, which have resulted in a high-quality paper that is ready for publication.

Reviewer #2: The authors present a clear and interesting study of how PrEP is understood among gay, bisexual, and other men who have sex with men in Nairobi, Kenya. It is likely to be of interest to the journal readers. I am aware I am a 'new' reviewer on an already revised manuscript, so I have tried to focus my comments on the methodological components of this work. The authors may with to consider the following:

- p3: small typo, should be "age-driven"

- p3: would rephrase "casual and other risky sexual behaviours", as implies 'casual' sex is inherently high risk for HIV (which it is not)

- p5: would specify $10 refers to USD (as there are many different version of the dollar)

- p7: small typo, should be "they collectively form..."

- p7: the mention of interviews in Tanzania is a bit confusing; if I'm not mistaken they are not included in this analysis, so suggest removing this reference.

- p7: the authors repeatedly mention 'purposive sampling' but have not specified the nature. If efforts were made to maximise sociodemographic diversity, identify certain experiences, or similar, then I would specify this information. I think purposive sampling is very appropriate here, but would just have appreciated a bit more detail on this approach.

- p7: relatedly, the authors mention their sample size was selected as "sufficient to yield thematic saturation." I understand this concept was introduced following comments from a previous reviewer, but it is widely out-of-favour among qualitative methodologists. It is unlikely anything like 'saturation' could be achieved with 22 people, but either way justification of 'sample size' is a positivist tradition better suited to quantitative study. I suggest the authors remove this sentence entirely. If there is some kind of need to 'justify' the sample, I would focus on its diversity, novelty, exploratory nature, or some other feature more aligned with the paradigms of qualitative inquiry.

- p9: I would perhaps not say the data were "analyzed using NVivo" as the software cannot really do analysis (would rephrase as: "Transcripts were organized using NVivo..." or similar)

- p9: I will not suggest a total reimagining of the analysis, but I must point out the results as presented do not appear to be of a thematic analysis. Themes must contain some kind of higher-level organisation of a corpus, one that goes beyond simply presenting what participants say. The latter is not bad, but it is not thematic; it is also what it appears the authors have done, organising the data as answers to the main questions asked of participants (as defined on p8). I would, therefore, strongly encourage the authors to rename their analysis as 'qualitative description' or a similar technique more aligned with their approach (see, for example: https://pmc.ncbi.nlm.nih.gov/articles/PMC5703087/)

- General note: I suggest greater engagement with the quotations as presented. Typically, the authors present them as evidence of a particular point, but offer no follow-up including to situate them within a larger context. For example, I note the last quote from 'Norman' (p18) indicates there are no MSM-specific clinics in Mombasa where one can access PrEP, but in fact there are several! While I understand this is the 'truth' as understood by this participant, this point is not properly contextualised. Adding 1-2 sentences after this and similar quotes will help situate these data within a larger context.

- p21: While I agree criminalisation (as a structural expression of stigma) is likely to have played a role here, this point is not really supported by the data as presented. Is there a quotation that can better support this linkage? If not, I would perhaps rephrase the opening sentence of line 435, using 'discrimination' as a foundation with which to link to larger systems that oppress gay, bisexual, and other men who have sex with men in Kenya

- p23: Relatedly, the discussion on injectable PrEP is appropriate, but how does the existence of ongoing stigma (and clear preference for population-specific services) align with the federal government's focus on 'integration' as the preferred model for HIV prevention and management? As this is an important topic in the current discourse of Kenya's HIV health systems, 1-2 sentences on this point may be valuable.

**********

7. PLOS authors have the option to publish the peer review history of their article (what does this mean?). If published, this will include your full peer review and any attached files.

**Do you want your identity to be public for this peer review?**  If you choose “no”, your identity will remain anonymous but your review may still be made public.

For information about this choice, including consent withdrawal, please see our Privacy Policy.

Reviewer #1: **Yes:**  SANNI, Olaniyi Felix

Reviewer #2: No

**********

[NOTE: If reviewer comments were submitted as an attachment file, they will be attached to this email and accessible via the submission site. Please log into your account, locate the manuscript record, and check for the action link "View Attachments". If this link does not appear, there are no attachment files.]

-->Figure Resubmissions:

While revising your submission, we strongly recommend that you use PLOS’s NAAS tool (https://ngplosjournals.pagemajik.ai/artanalysis) to test your figure files. NAAS can convert your figure files to the TIFF file type and meet basic requirements (such as print size, resolution), or provide you with a report on issues that do not meet our requirements and that NAAS cannot fix.

After uploading your figures to PLOS’s NAAS tool - https://ngplosjournals.pagemajik.ai/artanalysis, NAAS will process the files provided and display the results in the "Uploaded Files" section of the page as the processing is complete. If the uploaded figures meet our requirements (or NAAS is able to fix the files to meet our requirements), the figure will be marked as "fixed" above. If NAAS is unable to fix the files, a red "failed" label will appear above. When NAAS has confirmed that the figure files meet our requirements, please download the file via the download option, and include these NAAS processed figure files when submitting your revised manuscript.

# Author comment: Author response to Decision Letter 2

**DOI:** 10.1371/journal.pgph.0005282.r004

28 Apr 2026

Attachment Submitted filename: Response to Reviewers C.K 2.docx

# Peer review: Decision Letter 2

**DOI:** 10.1371/journal.pgph.0005282.r005

**Authors:** Helen Howard, Jen Edwards, Katherine Kokkinias

19 May 2026

PGPH-D-25-02608R2

“At the end of the day I'd have to use a condom!”: A qualitative investigation of use of HIV pre-exposure prophylaxis among young gay and bisexual men in Nairobi, Kenya

PLOS Global Public Health

Dear Dr. Cheruiyot,

Thank you for submitting your manuscript to PLOS Global Public Health. After careful consideration, we feel that it has merit but does not fully meet PLOS Global Public Health’s publication criteria as it currently stands. Therefore, we invite you to submit a revised version of the manuscript that addresses the points raised during the review process.

==============================

The manuscript has been reassessed by two reviewers, and their comments are available below.

The reviewers had additional concerns that need attention. Primarily, they have concerns regarding the language around themes/subthemes used in the manuscript.

Could you please revise the manuscript to carefully address the concerns raised?

==============================

Please submit your revised manuscript by Jun 17 2026 11:59PM. If you will need more time than this to complete your revisions, please reply to this message or contact the journal office at globalpubhealth@plos.org. When you're ready to submit your revision, log on to https://www.editorialmanager.com/pgph/ and select the 'Submissions Needing Revision' folder to locate your manuscript file.

Please include the following items when submitting your revised manuscript:

- A letter that responds to each point raised by the editor and reviewer(s). You should upload this letter as a separate file labeled 'Response to Reviewers'.
- A marked-up copy of your manuscript that highlights changes made to the original version. You should upload this as a separate file labeled 'Revised Manuscript with Track Changes'.
- An unmarked version of your revised paper without tracked changes. You should upload this as a separate file labeled 'Manuscript'.

Guidelines for resubmitting your figure files are available below the reviewer comments at the end of this letter.

As the corresponding author, your ORCID iD is verified in the submission system and will appear in the published article. PLOS supports the use of ORCID, and we encourage all coauthors to register for an ORCID iD and use it as well. Please encourage your coauthors to verify their ORCID iD within the submission system before final acceptance, as unverified ORCID iDs will not appear in the published article. *Only*  the individual author can complete the verification step; PLOS staff *cannot*  verify ORCID iDs on behalf of authors.

We look forward to receiving your revised manuscript.

Kind regards,

Katherine Demi Kokkinias, Ph.D.

Staff Editor

PLOS Global Public Health

Journal Requirements:

If the reviewer comments include a recommendation to cite specific previously published works, please review and evaluate these publications to determine whether they are relevant and should be cited. There is no requirement to cite these works unless the editor has indicated otherwise.

Please review your reference list to ensure that it is complete and correct. If you have cited papers that have been retracted, please include the rationale for doing so in the manuscript text, or remove these references and replace them with relevant current references. Any changes to the reference list should be mentioned in the rebuttal letter that accompanies your revised manuscript. If you need to cite a retracted article, indicate the article’s retracted status in the References list and also include a citation and full reference for the retraction notice.

Additional Editor Comments (if provided):

[Note: HTML markup is below. Please do not edit.]

Reviewers' comments:

Reviewer's Responses to Questions

**Comments to the Author**

1. If the authors have adequately addressed your comments raised in a previous round of review and you feel that this manuscript is now acceptable for publication, you may indicate that here to bypass the “Comments to the Author” section, enter your conflict of interest statement in the “Confidential to Editor” section, and submit your "Accept" recommendation.

Reviewer #1: All comments have been addressed

Reviewer #2: All comments have been addressed

**********

2. Does this manuscript meet PLOS Global Public Health’s publication criteria? Is the manuscript technically sound, and do the data support the conclusions? The manuscript must describe methodologically and ethically rigorous research with conclusions that are appropriately drawn based on the data presented.

Reviewer #1: Yes

Reviewer #2: Yes

**********

3. Has the statistical analysis been performed appropriately and rigorously?

Reviewer #1: Yes

Reviewer #2: N/A

**********

4. Have the authors made all data underlying the findings in their manuscript fully available (please refer to the Data Availability Statement at the start of the manuscript PDF file)?

The PLOS Data policy requires authors to make all data underlying the findings described in their manuscript fully available without restriction, with rare exception. The data should be provided as part of the manuscript or its supporting information, or deposited to a public repository. For example, in addition to summary statistics, the data points behind means, medians and variance measures should be available. If there are restrictions on publicly sharing data—e.g. participant privacy or use of data from a third party—those must be specified.

Reviewer #1: Yes

Reviewer #2: No

**********

5. Is the manuscript presented in an intelligible fashion and written in standard English?

PLOS Global Public Health does not copyedit accepted manuscripts, so the language in submitted articles must be clear, correct, and unambiguous. Any typographical or grammatical errors should be corrected at revision, so please note any specific errors here.

Reviewer #1: Yes

Reviewer #2: Yes

**********

6. Review Comments to the Author

Please use the space provided to explain your answers to the questions above. You may also include additional comments for the author, including concerns about dual publication, research ethics, or publication ethics. (Please upload your review as an attachment if it exceeds 20,000 characters)

Reviewer #1: I commend the authors for the thorough and thoughtful revisions made to the manuscript. You addressed each of my comments with clarity and depth, and the additions, particularly the expanded methodological detail, justification of sample size, and restructuring of the discussion around the IMB model, have strengthened the manuscript considerably. The revisions enhance transparency, theoretical alignment, and overall coherence. I appreciate the care and responsiveness demonstrated throughout this process.

Reviewer #2: I thank the authors for attending so carefully to my feedback. One final point is that the manuscript still repeatedly mentions ‘themes’ and ‘sub-themes’ despite the analytic shift away from thematic analysis. To avoid confusing the reader or inadvertently suggesting this was a thematic analysis, I would remove all such references and replace them with ‘categories’, ‘topics’, or similar. The idea of a qualitative descriptive analysis of this kind is that the data are organised around explicit questions or topics; instead of saying “…following sections explore the main themes that were defined from analysis”, it is probably more true to say “…following sections are organised around the primary topics explored through our interviews”. Could draw upon the IBM framework to give this shape. Once this final issue of semantics is resolved, I have no further feedback.

**********

7. PLOS authors have the option to publish the peer review history of their article (what does this mean?). If published, this will include your full peer review and any attached files.

**Do you want your identity to be public for this peer review?**  If you choose “no”, your identity will remain anonymous but your review may still be made public.

For information about this choice, including consent withdrawal, please see our Privacy Policy.

Reviewer #1: **Yes:**  Olaniyi Felix SANNI

Reviewer #2: No

**********

[NOTE: If reviewer comments were submitted as an attachment file, they will be attached to this email and accessible via the submission site. Please log into your account, locate the manuscript record, and check for the action link "View Attachments". If this link does not appear, there are no attachment files.]

Figure Resubmissions:

While revising your submission, we strongly recommend that you use PLOS’s NAAS tool (https://ngplosjournals.pagemajik.ai/artanalysis) to test your figure files. NAAS can convert your figure files to the TIFF file type and meet basic requirements (such as print size, resolution), or provide you with a report on issues that do not meet our requirements and that NAAS cannot fix.

After uploading your figures to PLOS’s NAAS tool - https://ngplosjournals.pagemajik.ai/artanalysis, NAAS will process the files provided and display the results in the "Uploaded Files" section of the page as the processing is complete. If the uploaded figures meet our requirements (or NAAS is able to fix the files to meet our requirements), the figure will be marked as "fixed" above. If NAAS is unable to fix the files, a red "failed" label will appear above. When NAAS has confirmed that the figure files meet our requirements, please download the file via the download option, and include these NAAS processed figure files when submitting your revised manuscript.

# Author comment: Author response to Decision Letter 3

**DOI:** 10.1371/journal.pgph.0005282.r006

17 Jun 2026

Attachment Submitted filename: Response_to_Reviewers_C.K_auresp_3.docx

# Editor report: Decision Letter 3

**DOI:** 10.1371/journal.pgph.0005282.r007

**Authors:** Helen Howard, Jen Edwards, Katherine Kokkinias, Julia Robinson

25 Jun 2026

“At the end of the day I'd still have to use a condom!”: A qualitative investigation of use of HIV pre-exposure prophylaxis among young gay and bisexual men in Nairobi, Kenya

PGPH-D-25-02608R3

Dear Mr. Cheruiyot,

We are pleased to inform you that your manuscript '“At the end of the day I'd still have to use a condom!”: A qualitative investigation of use of HIV pre-exposure prophylaxis among young gay and bisexual men in Nairobi, Kenya' has been provisionally accepted for publication in PLOS Global Public Health.

Before your manuscript can be formally accepted you will need to complete some formatting changes, which you will receive in a follow up email. A member of our team will be in touch with a set of requests.

Please note that your manuscript will not be scheduled for publication until you have made the required changes, so a swift response is appreciated.

IMPORTANT: The editorial review process is now complete. PLOS will only permit corrections to spelling, formatting or significant scientific errors from this point onwards. Requests for major changes, or any which affect the scientific understanding of your work, will cause delays to the publication date of your manuscript.

If your institution or institutions have a press office, please notify them about your upcoming paper to help maximize its impact. If they'll be preparing press materials, please inform our press team as soon as possible -- no later than 48 hours after receiving the formal acceptance. Your manuscript will remain under strict press embargo until 2 pm Eastern Time on the date of publication. For more information, please contact globalpubhealth@plos.org.

Thank you again for supporting Open Access publishing; we are looking forward to publishing your work in PLOS Global Public Health.

Best regards,

Julia Robinson

Executive Editor

PLOS Global Public Health

***********************************************************

Reviewer Comments (if any, and for reference):
